# Genetic variation in Tanis was associated with elevating plasma triglyceride level in Chinese nondiabetic subjects

**DOI:** 10.1186/1476-511X-12-97

**Published:** 2013-07-05

**Authors:** Ying Gao, Xiang Xie, Yi-Tong Ma, Yi-Ning Yang, Xiao-Mei Li, Zhen-Yan Fu, Ying-Ying Zheng, Xiang Ma, Bang-Dang Chen, Fen Liu, Ying Huang

**Affiliations:** 1 Department of Cardiology, First Affiliated Hospital of Xinjiang Medical University, Urumqi 830011, P.R. China; 2 Xinjiang Key Laboratory of Cardiovascular Disease Research, Urumqi 830011, P.R. China

**Keywords:** Genetics, Tanis, Triglyceride, Diabetes, Polymorphisms

## Abstract

**Background:**

The association of genetic polymorphisms of Tanis with triglyceride concentration in human has not been thoroughly examined. We aimed to investigate the relationship between triglyceride concentrations and Tanis genetic polymorphisms.

**Methods:**

All participants (n=1497) selected from subjects participating in the Cardiovascular Risk Survey (CRS) study were divided into two groups according to ethnicity (Han: n=1059; Uygur: n= 438). Four tagging SNPs (rs12910524, rs1384565, rs2101171, rs4965814) of Tanis gene were genotyped using TaqMan® assays from Applied Biosystems following the manufacturer’s suggestions and analyzed in an ABI 7900HT Fast Real-Time PCR System.

**Results:**

We found that the SNP rs12910524 was associated with triglyceride levels by analyses of a dominant model (*P*<0.001), recessive model (*P* <0.001) and additive model (*P* < 0.001) not only in Han ethnic but also in Uygur ethnic group, and the difference remained significant after the adjustment of sex, age, alcohol intake, smoking, BMI and plasma glucose (GLU) level (All *P* < 0.001). However, this relationship was not observed in rs1384565, rs2101171, and rs4965814 before and after multivariate adjustment (All P > 0.05). Furthermore, there were significant interactions between rs12910524 and GLU on TG both in Han (P=0.001) and Uygur population (P=2.60×10^-4^).

**Conclusion:**

Our results indicated that the rs12910524 in the *Tanis* gene was associated with triglyceride concentrations in subjects without diabetes in China.

## Background

Elevating triglycerides (TG) level, an essential component of the metabolic syndrome, is independently associated with coronary artery disease (CAD) [1]. High levels of fasting plasma TG are caused by not only environmental factors such as smoking[2-4], high-fat diet and alcohol intake [5,6], but also genetic factors including single nucleotide polymorphisms (SNPs). However, till date, only several candidate genes involving lipid metabolism [7-10] and CAD [11-14] have been discovered, and these genes only explain a small fraction of the total interindividual variation in plasma TG levels [15-17].

Tanis, a novel discovered membrane protein, has been suggested to be involved in the development of diabetes and dyslipidemia [18,19]. In a polygenic animal model of type 2 diabetes model-Psammomys obesus, the Tanis was found to be positively correlated to circulating TG concentrations [19]. However, the association of genetic polymorphisms of Tanis with plasma TG concentration in humans has not been thoroughly examined. In addition, Tanis was identified as a newly found receptor of amyloid A-1 (SAA1), which is not only an inflammatory marker but also an apolipoprotein [20]. In the previous study [20,21], we found that SAA1 gene polymorphisms were associated with dyslipidemia in Chinese subjects. Tanis, as a receptor of SAA1, also called SELS, located on chromosome15q26.3, encodes selenoprotein S which participates in the retro-translocation of misfolded proteins from the endoplasmic reticulum (ER) to the cytosol for their degradation [22]. Several previous studies indicated that the variations in Tanis gene were associated with pro-inflammatory cytokines such as interleukin (IL)-6, IL-1β and TNF-α [23] and cardiovascular disease [24] and metabolic factors [25]. However, the relationships between Tanis gene and lipid profile have not been thoroughly investigated. Xinjiang is part of the ancient Silk Road and borders eight countries including Russia, Kazakhstan, Kirghizastan, Tajikistan, Pakistan, Mongolia, India, and Afghanistan. There are more than 13 ethnic groups living in this area. Among them, the Uygur people account for 46%, and Han account for 40%. In this study, we aimed to observe the associations of tagging SNPs in Tanis gene with fasting plasma TG levels in Chinese Han and Uygur population in Xinjiang, the western China.

## Results and discussion

This study consists of two ethnic groups (Han: n=1059; Uygur: n= 438). The clinical and metabolic characteristics of the study population are shown separately for Han and Uygur in Table 1.

**Table 1:** Demographic and risk profile of the study population

**Risk factors**	**No. (%) or Mean±SD**		**P values**
	**Han (n=1059)**	**Uygur (n=438)**	
Age (years)	60.38 ± 11.81	63.16 ± 10.70	<0.001
Female (%)	481 (45.4)	174 (39.7)	0.043
Never drink (%)	837 (79.0)	393 (89.7)	<0.001
Former drinker (%)	201(19.0)	24 (5.5)	
Current drinker (%)	21 (2.0)	21 (4.8)	
Never smoking (%)	689 (65.1)	331 (75.6)	<0.001
Former smoking (%)	298 (28.1)	72 (16.4)	
Current smoking (%)	72 (6.8)	35 (8.0)	
BMI (Kg/m^2^)	24.52 ± 3.40	24.99 ± 3.99	0.020
SBP (mmHg)	122.21 ± 13.11	120.64 ± 10.07	0.025
DBP (mmHg)	76.75 ± 10.66	73.46 ± 7.40	<0.001
GLU (mmol/L)	4.57 ± 0.86	4.30 ± 0.45	<0.001
TG (mmol/L)	0.96 ± 0.34	0.93 ± 0.35	0.080
TC (mmol/L)	4.26 ± 0.98	4.12 ± 0.94	0.015
HDL (mmol/L)	1.28 ± 0.44	1.27 ± 0.47	0.172
LDL-C (mmol/L)	2.65 ± 0.81	2.54 ± 0.80	0.016

Note: *HDL* high-density lipoprotein, *LDL* low-density lipoprotein, *SBP* Systolic blood pressure, *DBP* Diastolic blood pressure, *TG* Triglycerides, *TC* Cholesterol, *BMI* Body mass index, *GLU* Glucose.

All genotyped SNPs were in Hardy-Weinberg equilibrium (all *P*>0.05, data not shown). Table 2 shows detailed information for each SNP as well as the allele frequencies.

**Table 2:** Distributions of SNPs of Tanis gene in Han and Uygur population

**SNPs**	**Genotypes**	**Ethnic**		**P value**
		**Han, n (%)**	**Uygur, n (%)**	
rs12910524	TT	158 (14.9)	62 (14.2)	0.406
	TC	486 (45.9)	188 (42.9)	
	CC	415 (39.2)	188 (42.9)	
rs1384565	CC	74 (7.0)	11 (2.5)	<0.001
	CT	442 (41.7)	102 (23.3)	
	TT	543 (51.3)	325 (74.2)	
rs2101171	CC	30 (2.8)	10 (2.3)	<0.001
	CT	320 (30.2)	109 (24.9)	
	TT	709 (66.9)	319 (72.8)	
rs4965814	CC	182 (17.2)	68 (15.5)	0.034
	CT	517 (48.8)	190 (43.4)	
	TT	360 (34.0)	180 (41.1)	

Both in Chinese Han and Uygur populations, we found that the rs12910524 was significantly associated with plasma TG levels in a dominant model, additive model, or recessive model before (All P <0.001) and after multivariate adjustment (All P <0.001; Table 3). However, these associations were not found in rs1384565, rs2101171, and rs4965814 before and after adjustment of confounders. Furthermore, using the general linear model analysis, we found that the GLU level was significantly associated with TG level both in Han (P=0.001) and Uygur populations (P=2.99×10^-6^). And, we also found significant interactions between rs12910524 and GLU on plasma TG both in Han (P=0.012; Table 4) and Uygur populations (P=2.60×10^-4^; Table 5). However, we did not find any interaction between rs1384565, rs2101171, and rs4965814 and GLU level (Table 4, Table 5).

**Table 3:** **Association of *Tanis *SNPs with log-transformed TG value in Han and Uygur population**

		**Mean log-transformed TG level**			**Model 1‡**			**Model 2§**		
	**Wild/rare allele**	**Homozygous for rare allele**	**Heterozygous**	**Homozygous for wild allele**	*P *Rec*	***P *Dom†**	***P *Add**^**※**^	*P *Rec*	***P *Dom†**	***P *Add**^**※**^
Han										
rs12910524	C/T	-0.32 ± 0.45	-0.11 ± 0.39	-0.02 ± 0.33	**<0.001**	**<0.001**	**<0.001**	**<0.001**	**<0.001**	**<0.001**
rs1384565	T/C	-0.11 ± 0.46	-0.12 ± 0.38	-0.98 ± 0.39	0.337	0.955	0.595	0.222	0.967	0.452
rs2101171	T/C	-0.26 ± 0.41	-0.09 ± 0.40	-0.11 ± 0.38	0.902	0.038	0.094	0.864	0.019	0.048
rs4965814	T/C	-0.09 ± 0.41	-0.12 ± 0.38	-0.10 ± 0.39	0.649	0.585	0.688	0.509	0.492	0.510
Uygur										
rs12910524	C/T	-0.50 ± 0.49	-0.15 ± 0.41	-0.05 ± 0.32	**<0.001**	**<0.001**	**<0.001**	**<0.001**	**<0.001**	**<0.001**
rs1384565	T/C	-0.30 ± 0.53	-0.16 ± 0.45	-0.15 ± 0.40	0.547	0.233	0.471	0.485	0.212	0.431
rs2101171	T/C	-0.17 ± 0.41	-0.12 ± 0.42	-0.14 ± 0.47	0.928	0.272	0.537	0.778	0.295	0.494
rs4965814	T/C	-0.23 ± 0.43	-0.15 ± 0.43	-0.13 ± 0.39	0.351	0.097	0.237	0.366	0.066	0.179

§Analysis of covariance adjusted for sex, age, smoking, alcohol drinking, and GLU; ‡Unadjusted model; *recessive model; †dominant model; ^※^additive model.

**Table 4:** Interactions between SNPs of Tanis and GLU on TG levels in Chinese Han population

**Source**	**Type III sum of squares**	**df**	**Mean square**	**F**	**Sig.**
Corrected model	15.139^a^	13	1.165	8.322	3.50×10^-16^
Age	0.051	1	0.051	0.365	0.546
Sex	0.030	1	0.030	0.216	0.642
Smoking	0.012	1	0.012	0.085	0.771
Drinking	0.117	1	0.117	0.838	0.360
BMI	0.028	1	0.028	0.202	0.653
rs12910524	2.036	2	1.018	7.273	**0.001**
GLU * rs12910524	0.933	3	0.311	2.222	**0.012**
GLU * rs4965814	0.283	1	0.283	2.021	0.155
GLU * rs2101171	0.232	1	0.232	1.658	0.198
GLU * rs1384565	0.312	1	0.312	2.227	0.136
Error	146.240	1045	0.140		
Total	173.988	1059			
Corrected total	161.380	1058			

^a^R Squared = 0.094 (Adjusted R Squared =0.083).

**Table 5:** Interactions between SNPs of Tanis and GLU on TG levels in Chinese Uygur population

**Source**	**Type III sum of squares**	**df**	**Mean square**	**F**	**P value**
Corrected model	16.946^a^	13	1.304	9.513	1.97×10^-17^
Age	0.132	1	0.132	0.965	0.326
sex	0.011	1	0.011	0.079	0.779
Smoking	0.001	1	0.001	0.010	0.920
Drinking	0.240	1	0.240	1.748	0.187
BMI	0.419	1	0.419	3.061	0.081
rs12910524	3.593	2	1.796	13.109	**2.99×10**^**-6**^
GLU * rs12910524	2.675	3	0.892	6.507	**2.60×10**^**-4**^
GLU * rs4965814	0.360	1	0.360	2.630	0.106
GLU * rs2101171	0.148	1	0.148	1.083	0.299
GLU * rs1384565	0.147	1	0.147	1.074	0.301
Error	58.103	424	0.137		
Total	85.395	438			
Corrected total	75.049	437			

^a^R Squared =0.226 (Adjusted R Squared = 0.202).

In Chinese Uygur population, we found that the rs12910524 was significantly associated with plasma TC levels in a dominant model, additive model, or recessive model before (All P <0.01) and after multivariate adjustment (All P <0.01; Table 6). And we also found that the rs12910524 was significantly associated with plasma LDL-C level in a recessive model and an additive model before (All P <0.01) and after multivariate adjustment (All P <0.01; Table 7). In addition, we found the rs1384565 was significantly associated with plasma HDL-C level in a dominant model and in an additive model after multivariate adjustment (both P<0.01; Table 8). However, we did not find any association of Tanis genetic polymorphisms with plasma TC, HDL-C, and LDL-C levels in Chinese Han population.

**Table 6:** **Association of *Tanis *SNPs with TC in Han and Uygur population**

		**Mean TC level**			**Model 1‡**			**Model 2§**		
	**Wild/Rare allele**	**Homozygous for rare allele**	**Heterozygous**	**Homozygous for wild allele**	*P *Rec*	***P *Dom†**	***P *Add**^**※**^	*P *Rec*	***P *Dom†**	***P *Add**^**※**^
Han										
rs12910524	C/T	4.11 ± 1.13	4.24 ± 0.95	4.34 ± 0.92	0.047	0.034	0.042	0.062	0.107	0.10
rs1384565	T/C	4.26 ± 1.01	4.30 ± 0.99	4.22 ± 0.99	0.265	0.998	0.510	0.493	0.761	0.692
rs2101171	T/C	4.09 ± 1.01	4.24 ± 0.95	4.26 ± 0.97	0.479	0.330	0.553	0.529	0.142	0.328
rs4965814	T/C	4.17 ± 1.03	4.29 ± 0.98	4.26 ± 0.92	0.955	0.184	0.362	0.757	0.274	0.549
Uygur										
rs12910524	C/T	3.62 ± 0.99	4.13 ± 0.93	4.28 ± 0.89	**<0.001**	**0.003**	**<0.001**	**<0.001**	**0.005**	**<0.001**
rs1384565	T/C	3.59 ± 1.19	4.15 ± 0.93	4.13 ± 0.94	0.731	0.058	0.163	0.655	0.079	0.213
rs2101171	T/C	4.13 ± 0.94	4.06 ± 0.94	4.38 ± 1.05	0.391	0.622	0.530	0.498	0.535	0.570
rs4965814	T/C	4.08 ± 1.03	4.09 ± 0.97	4.18 ± 0.88	0.310	0.689	0.597	0.361	0.526	0.625

§Analysis of covariance adjusted for sex, age, smoking, alcohol drinking, and GLU; ‡Unadjusted model; *recessive model; †dominant model; ^※^additive model.

**Table 7:** **Association of *Tanis *SNPs with LDL-C in Han and Uygur population**

		**Mean LDL-C level**			**Model 1‡**			**Model 2§**		
	**Wild/Rare allele**	**Homozygous for rare allele**	**Heterozygous**	**Homozygous for wild allele**	*P *Rec*	***P *Dom†**	***P *Add**^**※**^	*P *Rec*	***P *Dom†**	***P *Add**^**※**^
Han										
rs12910524	C/T	2.55 ± 0.93	2.61 ± 0.79	2.72 ± 0.77	0.091	0.014	0.032	0.126	0.034	0.071
rs1384565	T/C	2.71 ± 0.90	2.70 ± 0.82	2.60 ± 0.78	0.043	0.502	0.128	0.119	0.731	0.295
rs2101171	T/C	2.38 ± 0.80	2.60 ± 0.74	2.68 ± 0.83	0.051	0.069	0.057	0.052	0.036	0.037
rs4965814	T/C	2.55 ± 0.84	2.68 ± 0.82	2.65 ± 0.77	0.970	0.070	0.163	0.869	0.151	0.339
Uygur										
rs12910524	C/T	2.25 ± 0.80	2.52 ± 0.81	2.64 ± 0.78	**0.003**	0.022	**0.004**	**0.005**	0.025	**0.007**
rs1384565	T/C	2.02 ± 0.84	2.52 ± 0.78	2.56 ± 0.81	0.312	0.032	0.091	0.323	0.036	0.102
rs2101171	T/C	2.54 ± 0.79	2.53 ± 0.86	2.57 ± 0.80	0.905	0.951	0.988	0.888	0.945	0.990
rs4965814	T/C	2.40 ± 0.47	2.55 ± 0.82	2.58 ± 0.80	0.395	0.117	0.280	0.447	0.106	0.267

§Analysis of covariance adjusted for sex, age, smoking, alcohol drinking, and GLU; ‡Unadjusted model; *recessive model; †dominant model; ^※^additive model.

**Table 8:** **Association of *Tanis *SNPs with HDL-C in Han and Uygur population**

		**Mean HDL-C level**			**Model 1‡**			**Model 2§**		
	**Wild/Rare allele**	**Homozygous for rare allele**	**Heterozygous**	**Homozygous for wild allele**	*P *Rec*	***P *Dom†**	***P *Add**^**※**^	*P *Rec*	***P *Dom†**	***P *Add**^**※**^
Han										
rs12910524	C/T	1.28 ± 0.41	1.32 ± 0.44	1.30 ± 0.46	0.428	0.607	0.518	0.505	0.388	0.414
rs1384565	T/C	1.32 ± 0.54	1.31 ± 0.45	1.30 ± 0.42	0.499	0.772	0.792	0.516	0.682	0.787
rs2101171	T/C	1.27 ± 0.52	1.31 ± 0.44	1.31 ± 0.44	0.996	0.673	0.910	0.974	0.470	0.752
rs4965814	T/C	1.27 ± 0.47	1.31 ± 0.44	1.32 ± 0.43	0.442	0.215	0.430	0.289	0.186	0.335
Uygur										
rs12910524	C/T	1.19 ± 0.42	1.24 ± 0.40	1.33 ± 0.54	0.127	0.025	0.059	0.285	0.065	0.062
rs1384565	T/C	1.64 ± 1.72	1.31 ± 0.34	1.25 ± 0.40	0.073	**0.007**	0.014	0.103	**0.002**	**0.007**
rs2101171	T/C	1.28 ± 0.50	1.22 ± 0.35	1.51 ± 0.51	0.096	0.498	0.131	0.341	0.347	0.126
rs4965814	T/C	1.39 ± 0.77	1.25 ± 0.38	1.25 ± 0.39	0.413	0.025	0.082	0.424	0.047	0.193

§Analysis of covariance adjusted for sex, age, smoking, alcohol drinking, and GLU; ‡Unadjusted model; *recessive model; †dominant model; ^※^additive model.

In this study, we observed that variation in the Tanis gene was associated with plasma TG levels in Chinese subjects. Individuals with the C allele of rs12910524 had significantly higher plasma TG levels when compared with TT genotype carriers. To our knowledge, this is the first study to investigate the common allelic variant in Tanis gene and its association with plasma TG levels.

The human Tanis gene is located at 15q26.3. Although this region has not been previously identified in genome-wide linkage scans for diabetes-related phenotypes in human populations, previous studies [25] indicated that the Tanis gene expression was positively correlated to BMI, plasma levels of TG and HDL cholesterol, insulin, and blood glucose levels. Also, several studies suggested that the variations in Tanis gene were associated with inflammation [23], coronary heart disease (CHD) and ischemic stroke [24], and metabolic disease [25].

The plasma triglyceride level is known to be influenced by a large number of factors, including age, sex, hypertension, diabetes, smoking and alcohol intake. Our findings show that rs1291054 is an independent determinant of triglyceride level, and does not influence the level by modulating some confounding factors such age, sex, smoking, BMI, and alcohol intake. Walder et al. [19] described the biological characteristics of Tanis first. In their study, they found that Tanis gene expression was increased 2.2-fold after a 24-h fast in *P. obesus,* a polygenic animal model of type 2 diabetes and metabolic syndrome. Also, they found that there was a positive correlation between Tanis expression and circulating TG concentrations (Pearson r = 0.593, P = 0.007); as well as blood glucose (Spearman r = 0.378, P = 0.010) and insulin concentrations (Spearman r = 0.416, P = 0.004). However, subsequently multiple linear regression analysis indicated that only the change in blood glucose concentration was independently associated with Tanis gene expression. This result suggests that the association of Tanis gene expression with TG level can be modified by blood glucose level. Therefore, in this study, we excluded the diabetic patients when we selected participants at the beginning of the study, and we found that in nondiabetic subjects, the rs12910524 was independently associated with plasma TG level, and this relationship was not modified by the fasting blood glucose level. And this association was observed not only in Chinese Han but also in Chinese Uygur population. We also analyzed the associations of Tanis genetic polymorphisms with plasma TC, HDL-C, and LDL-C levels. In Chinese Uygur population, we found that the rs12910524 was significantly associated with plasma TC levels and plasma LDL-C levels. And, we also found that the rs1384565 was significantly associated with plasma HDL-C levels. However, we did not find any association of Tanis genetic polymorphisms with plasma TC, HDL-C, and LDL-C levels in Chinese Han population. This discrepancy may be explained by the different distributions of Tanis genetic polymorphisms and some confounders between Chinese Han and Uygur population.

In addition, some published data indicated that inflammatory genes may regulate fasting TG levels [26]. And previous studies also indicated that Tanis gene was associated with inflammatory cytokines [23]. In the present study, we found Tanis genetic polymorphism was associated with TG level. However we have no evidences to demonstrate whether this association was related to inflammation because of the absence of some inflammatory cytokines parameters. Otherwise, because of the absence of some confounders such as plasma HOMA-IR or HbA1c levels, eating habits, working pressure and the social disparities in our database, we did not include these variables in the multivariate analysis. This fact is a limitation of our study.

## Conclusions

In conclusion, our results indicate that the Tanis gene rs12910524 polymorphism is an important and clinically relevant determinant of plasma TG levels in the Chinese subjects without diabetes.

## Subjects and methods

### Subjects

This study was approved by the Ethics Committee of the First Affiliated Hospital of Xinjiang Medical University and was conducted according to the standards of the Declaration of Helsinki. Written informed consent was obtained from the participants. All the participants were selected from the Cardiovascular Risk Survey (CRS) study which was described in the previous studies [27,28]. From these subjects participating in CRS (n=14 618), we selected 1821 participants who were free from diabetes, hypertension, any history of CAD, or any history of taking lipid-lowering drugs. We defined diabetes by using the American Diabetes Association (ADA) 2009 criteria as described previously [29] (fasting plasma glucose ≥7.0 mmol/L [≥126 mg/dL]) or self-reported current diabetes treatments in the survey. Among these 1821 participants, only 1740 (Han: n= 1251; Uygur: n= 489) participants consented to providing blood samples for DNA analysis. We excluded 243 hypertriglyceridemia (fasting plasma TG ≥1.7 mmol/L) patients during the analysis. The analysis presented in this study was based on 1 497 subjects (Han: n= 1059; Uygur: n= 438) who had passed the eligibility criteria and had complete data on Tanis genotype.

### Biological and lifestyle measurements

Height, body weight, and blood pressure were measured as described previously [27,28]. Smoking and drinking status was self-reported by study questionnaire as described previously [27,28]. We measured the fasting plasma concentration of total cholesterol, triglyceride (TG), low-density lipoprotein (LDL), high-density lipoprotein (HDL) and glucose using an equipment for chemical analysis (Dimension AR/AVL Clinical Chemistry System, Newark, NJ) employed by the Clinical Laboratory Department of the First Affiliated Hospital of Xinjiang Medical University as described previously [27-31].

### Tanis single-nucleotide polymorphism genotyping

There are 190 SNPs for the human Tanis gene listed in the National Center for Biotechnology Information SNP database (http://www.ncbi.nlm.nih.gov/ SNP).

We also screened the data for the Tag SNPs on the International HapMap Project website (http://www.hapmap.org/). Using the Haploview 4.2 software and the HapMap phrase II database, we obtained four tagging SNPs (rs12910524, rs1384565, rs2101171, and rs4965814) for Chinese Han using minor allele frequency (MAF) ≥ 0.05 and linkage disequilibrium patterns with r^2^ ≥ 0.8 as a cutoff.

Genomic DNA was extracted from the peripheral blood leukocytes using a DNA extraction Kit (Beijing Bioteke Co. Ltd, China). Genotyping was confirmed using TaqMan® assays from Applied Biosystems following the manufacturer’s suggestions and analyzed in an ABI 7900HT Fast Real-Time PCR System. To ensure the results to be verified, of the genotyped samples, 10% were duplicated and there was at least one positive and one negative control per 96-well DNA plate in our assays. The accuracy of the genotyping was determined by the genotype concordance between duplicate samples. We obtained a 100% concordance between the genotyped duplicate samples.

### Statistical analysis

All analyses were carried out using SPSS version 17.0 (SPSS Inc., Chicago, IL, USA). The Hardy-Weinberg equilibrium was assessed using chi-square analysis. The characteristics of the study population were expressed as the mean ± standard deviation or as a ratio. Fasting triglycerides were log-transformed using natural logarithms for analysis. General linear model analysis was undertaken to test for associations between SNP genotypes and TG levels after adjusting for confounding variables. Single-SNP effects with continuous variables were analyzed using linear regression using three models. These were the additive (common allele homozygotes coded as 1, heterozygotes as 2, and recessive allele homozygotes as 3); dominant (common allele homozygotes coded as 1 and heterozygotes and recessive allele homozygotesas 2); and recessive (common allele homozygotes and heterozygotes coded as1 and recessive allele homozygotes as 2) models as described previously [16]. Normality was assessed by plotting the residuals. To assess the association of each SNP with TG level, we used a Bonferroni correction to control for the number of variants tested; this was 4, so the probability value, 0.0125, was considered to be significant.

## Abbreviations

SNP: Single nucleotide polymorphisms; CAD: Coronary artery disease; SAA: Serum amyloid A; TG: Triglycerides; TC: Total cholesterol; HDL-C: High-density lipoprotein; LDL-C: Low-density lipoprotein.

## Competing interests

The authors declare that they have no competing interests.

## Authors’ contributions

YG and XX carried out the molecular genetic studies and drafted the manuscript.YNY, ZYF and XML carried out the genotyping. XM, YC, and BDC participated in the design of the study and performed the statistical analysis. YTM, YH, FL and YYZ conceived of the study and participated in its design and coordination and helped to draft the manuscript. All authors read and approved the final manuscript.
